# Peripheral Thyroid Hormones, Inflammatory and Skeletal Muscle Indexes in Advanced Cervical Cancer Treated With Cemiplimab

**DOI:** 10.1002/jcsm.70101

**Published:** 2025-10-13

**Authors:** Valentina Tuninetti, Elisa Virano, Amedeo Calvo, Vittoria Carbone, Carmela Pisano, Monika Ducceschi, Giacinto Turitto, Giuseppa Scandurra, Maria Cristina Petrella, Valeria Forestieri, Massimo Petracchini, Alessandra Bianco, Raffaella Cioffi, Mara Mantiero, Eleonora Paluzzi, Maria Grazia Distefano, Olga Martelli, Sandro Pignata, Vincenzo Formica, Fotios Loupakis, Giorgio Valabrega

**Affiliations:** ^1^ Department of Oncology, University of Turin, Medical Oncology Ordine Mauriziano Hospital Turin Italy; ^2^ Department of Oncology University of Turin Turin Italy; ^3^ Department of Diagnostic and Interventional Radiology Ordine Mauriziano Hospital Turin Italy; ^4^ Department of Woman, Child and Public Health Fondazione Policlinico Universitario a. Gemelli IRCCS Rome Italy; ^5^ Istituto Nazionale Tumori di Napoli Fondazione G Pascale IRCCS Naples Italy; ^6^ Fondazione IRCCS Istituto Nazionale Dei Tumori di Milano Italy; ^7^ Division of Oncology AORN “Sant' Anna e San Sebastiano” Caserta Italy; ^8^ Medical Oncology Unit Cannizzaro Hospital Catania Italy; ^9^ Oncologia Medica Ginecologica Azienda Universitaria Ospedaliera Careggi Florence Italy; ^10^ UOC Oncologia Medica Azienda Ospedaliera Universitaria Federico II Naples Italy; ^11^ Department of Hospital Pharmacy Ordine Mauriziano Hospital Turin Italy; ^12^ Obstetrics and Gynecology Unit IRCCS San Raffaele Scientific Institute Milan Italy; ^13^ School of Medicine Vita‐Salute San Raffaele University Milan Italy; ^14^ Medical Oncology Unit, Department of Systems Medicine Tor Vergata University Rome Italy; ^15^ 3trees Healthcare Viterbo Italy; ^16^ KISS No profit Association Pisa Italy

**Keywords:** cemiplimab, cervical cancer, fT3/fT4 ratio, MITO 44 study, prognosis, skeletal muscle index, systemic inflammatory index, thyroid hormones

## Abstract

**Background:**

A low fT3/fT4 ratio has been associated with poorer prognosis in several diseases. Inflammatory indexes (IIs) and the skeletal muscle index (SMI) are established prognostic factors in various cancer types. However, their interplay and individual contributions to the prognosis of cervical cancer remain unclear. This study aimed to evaluate the impact of these biomarkers on survival outcomes in cervical cancer patients treated with innovative immunotherapy.

**Methods:**

This retrospective study included 101 patients with cervical cancer treated with cemiplimab at 12 Italian oncology centres. Patients with thyroid comorbidities or missing fT3/fT4 ratio data were excluded. The primary endpoint was overall survival (OS) in relation to the fT3/fT4 ratio. Secondary endpoints included progression‐free survival (PFS) and correlations between the fT3/fT4 ratio, ECOG Performance Status, IIs and SMI.

**Results:**

An optimal fT3/fT4 cutoff for OS prediction was identified at 0.29. Median OS was 10.9 months for patients with a low fT3/fT4 ratio, while it was not reached for those with high fT3/fT4 levels (HR = 2.70; 95% CI: 1.17–6.22; *p* = 0.02). Multivariate analysis confirmed that both the fT3/fT4 ratio and ECOG PS independently influenced OS. Among the IIs analysed, the systemic inflammatory index (SII) demonstrated the strongest correlation with fT3/fT4 levels (OR = 3.82; 95% CI: 1.39–10.50; *p* = 0.0092). Exploratory analysis also revealed significantly lower SMI values in patients with lower fT3/fT4 ratios (*p* = 0.034).

**Conclusions:**

This study highlights the prognostic significance of the fT3/fT4 ratio, IIs, and SMI in cervical cancer patients treated with cemiplimab. Given the exploratory nature of these findings, further validation in larger, prospective cohorts is warranted to support their integration into clinical practice and the development of innovative prognostic tools.

## 1 Introduction

Cervical cancer (CC), the fourth most common cancer among women worldwide, has seen significant treatment advances with the advent of immunotherapy [1]. Immune checkpoint inhibitors (ICIs), particularly anti‐PD‐1/PD‐L1 antibodies, have demonstrated notable efficacy. Pembrolizumab was first approved as a second‐line treatment for advanced CC (aCC) [2] and later as a first‐line option for patients with a PD‐L1 combined positive score (CPS) ≥ 1 when combined with platinum‐based chemotherapy, with or without bevacizumab [3]. Recent trials have further expanded ICI's use: Pembrolizumab combined with chemo‐radiotherapy improved overall survival (OS) in locally aCC (ENGOT‐cx11/GOG‐3047/KEYNOTE‐A18) [4]; atezolizumab added to bevacizumab plus platinum significantly enhanced progression‐free and OS in the BEAT‐cc trial [5]. Cemiplimab, an IgG4 monoclonal antibody against PD‐L1, improved both PFS and OS versus chemotherapy in the phase III EMPOWER‐CERVICAL 1 trial [6] and in real‐world settings [7].

Clinical and molecular features such as ECOG performance status, tumour burden at diagnosis, previous number and lines of treatments, and PD‐L1 status have been identified as prognostic factors for aCC; however, new and better tools are required to guide patients’ selection for optimal treatment, for improving methods of patients’ stratification in clinical trials and data analyses in clinical and translational research [8].

Peripheral thyroid hormone conversion plays a pivotal role in regulating numerous physiological functions, including central metabolic processes. This conversion is primarily mediated by deiodinase enzymes, which catalyse the removal of iodine atoms from thyroxine (T4), producing the biologically active triiodothyronine (T3) and its metabolites. The activity of deiodinases is modulated by various factors, notably nutritional status and hormonal signals.

Over the past years, several studies have underscored the prognostic significance of the free triiodothyronine (fT3)/free thyroxine (fT4) ratio. In particular, a low fT3/fT4 ratio has emerged as a negative prognostic marker in several clinical conditions, including multiple solid tumours such as advanced metastatic colorectal cancer [9, 10], renal cell carcinoma [11], urothelial carcinoma [12] and hepatocellular carcinoma [13].

Because peripheral thyroid hormone activity is tightly linked to the fT3/fT4 ratio, which reflects the efficiency of peripheral conversion, it has been hypothesised that the enzymatic activity of deiodinase types 1 and 2 (D1 and D2) is modulated as a metabolic response to tumour burden and clinical aggressiveness [14]. Specifically, D1 is predominantly expressed in the liver and kidneys, where it catalyses the conversion of T4 to T3, potentially enhancing local thyroid signalling in various cancers. Conversely, D2, primarily active in the central nervous system and brown adipose tissue, contributes to local tissue‐specific regulation of thyroid hormone signalling, and its expression may also be upregulated in tumours, sustaining cancer progression.

To date, no studies have specifically explored the prognostic relevance of the fT3/fT4 ratio in patients with aCC.

Systemic inflammatory markers such as the immune inflammation index (SII), based on peripheral lymphocyte, neutrophil and platelet counts; the systemic inflammation response index (SIRI), based on peripheral neutrophil counts, monocytes counts and lymphocytes counts; and the ratio of neutrophils and lymphocytes (N/L) showed their prognostic values in various settings [15, 16, 17, 18]; however, evidence in metastatic/recurrence CC is limited. SII, SIRI and N/L have been considered good indexes that reflect the local immune response and systemic inflammation.

The relationship between cancer progression and clinical decline up to cancer cachexia and their causative and mutually worsening connection with systemic inflammation is well established.

Importantly, systemic inflammation correlates with low skeletal muscle index (SMI) independently of thyroid hormone levels. Pro‐inflammatory cytokines such as interleukin‐6 (IL‐6), tumour necrosis factor‐alpha (TNF‐α) and C‐reactive protein (CRP) promote muscle catabolism by activating proteolytic pathways and inhibiting muscle protein synthesis [19, 20]. This highlights inflammation as a key driver of sarcopenia beyond thyroid hormone activity. In CC patients, systemic inflammatory status has been linked to muscle wasting and poorer clinical outcomes, underscoring its prognostic significance beyond alterations in thyroid hormone metabolism [21].

Previous studies have shown that patients with sarcopenia have worse progression‐free survival (PFS) and OS rates [22, 23, 24]. Sarcopenia itself is emerging as an independent prognostic factor in various cancers, including CC, with patients exhibiting reduced PFS and OS. The interplay between skeletal muscle, immune function and chronic inflammation involves cytokines such as IL‐6, IL‐7 and IL‐15, which modulate immune responses. Conversely, chronic inflammation induces T cell exhaustion via cytokines like TGF‐β and IL‐10 and inhibitory receptors such as PD‐1 on CD8 + T cells, potentially diminishing the efficacy of ICIs, lowering these cytokines may weaken immune systems and reduce their ability to respond to malignancies [25].

Muscle wasting or sarcopenia, quantified by SMI via computed tomography (CT) at the third lumbar vertebra (L3), is now a standard measure for muscle mass assessment and is emerging as an independent prognostic indicator in patients with certain cancers [19] especially in head and neck tumours [20, 22]. Measurement of SMI using CT images in a single abdominal cross‐sectional image at the level of the third lumbar vertebra (L3) is a new standard in muscle mass assessment [26, 27, 28, 29, 30, 31, 32].

Numerous studies have demonstrated that reduced muscle mass at this anatomical landmark is associated with adverse clinical outcomes, including decreased treatment tolerance, increased therapy‐related toxicity and reduced OS in cancer patients. Specifically, Lee et al. reported that skeletal muscle depletion measured at L3 serves as an independent prognostic indicator of shorter PFS and OS in patients with locally aCC undergoing definitive chemoradiotherapy [31]. Similarly, Han et al. utilised an artificial intelligence‐driven volumetric approach to quantify sarcopenia on CT images, confirming the significant association between reduced muscle volume at L3 and poorer survival outcomes in early‐stage CC [30]. The selection of L3 as a standardised anatomical landmark is further supported by studies demonstrating strong quantitative and qualitative correlations of muscle mass between this vertebral level and others, validating its reliability as a surrogate for whole‐body muscle assessment [26, 29].

Nutritional status is related to both thyroid disorders and systemic inflammatory markers: Inflammation may impact the development and progression of thyroid function disorders, and some studies demonstrated that overt thyroid diseases are associated with decline in muscle mass and strength. Nutritional status plays a key role in cancer treatment and prognosis.

Correlation between those features and their independent contribution to prognosis of aCC is unclear.

We planned the present analysis of the MITO 44 study [7] to (a) investigate the prognostic role of the fT3/fT4 ratio in aCC patients treated with cemiplimab and to (b) assess the association between fT3/fT4 ratio and marker of inflammation or sarcopenia.

## 2 Materials and Methods

This retrospective cohort study included data from patients treated within the nominal use program in 12 Italian Multicenter Italian Trials in Ovarian Cancer and Gynaecological Malignancies (MITO) centres. As specified in the primary publication [7], data were collected from the patients' medical records using REDCap (research electronic data capture) v14.1.2. Inclusion/exclusion criteria were those of the EMPOWER‐CERVICAL‐1 trial. Inclusion criteria were age > 18 years and histologically confirmed diagnosis of recurrent, persistent or metastatic CC previous treated with platinum‐based chemotherapy, not eligible or able to participate in a clinical trial in this indication. Serum creatinine ≤ 1.5× ULN or estimated creatinine clearance > 45 mL/min, adequate bone marrow and liver function were even required. Exclusion criteria were patients with a history of solid organ transplant; ongoing or within 5 years autoimmune disease that requires immunosuppressive treatments; prior treatment with an anti‐PD (L)‐1 mAb (monoclonal antibody); prior treatment with other immune modulating agents that was within fewer than 28 days prior to the first dose of cemiplimab; presence of untreated brain metastasis that may be considered active; treatment with corticosteroid > 10 mg/day or equivalent) < 4 weeks prior to the first dose of cemiplimab; active infection (bacterial, viral, fungal or mycobacterial) requiring therapy, including infection with human immunodeficiency virus (HIV), or active infection with hepatitis B virus (HBV) or hepatitis C virus (HCV); receipt of live vaccine (including attenuated) within 30 days of first dose of cemiplimab; history of non‐infectious pneumonitis within the last 5 years; history of documented allergic reactions or acute hypersensitivity reaction attributed to mAb treatment, breast feeding, positive, serum pregnancy test and prior treatment with idelalisib. Common toxicity criteria (CTCAE), version 5.0, were applied to grade the side‐effects. All patients signed a written informed consent, and the study was approved by local ethics committees. Cemiplimab was administered at the dose of 350 mg flat dose in a 3‐week schedule. No dose reduction was allowed. Cemiplimab was continued as long as it was clinically appropriate, based on tumour assessment (until disease progression or inadequate therapeutic effect and in the presence of unacceptable side‐effects) and judgement of the treating physician. Disease evaluation was done as per clinical practice every 12 to 14 weeks with thorax and abdomen CT scans. Efficacy assessments were defined using RECIST 1.1 criteria. The timing of blood sample collection was within 7 days before treatment as for BMI calculation.

In this secondary study, of the 128 patients treated with cemiplimab, 27 were excluded due to thyroid comorbidities or lack of data on the fT3/fT4 ratio. Consequently, a total of 101 patients were included in the final analysis. The primary endpoint was OS in relation to the fT3/fT4 ratio. Secondary endpoints included the prognostic impact of ECOG Performance Status, SII, SIRI, N/L and SMI. Variables were categorised as follows: ECOG PS: 0–1 versus 2, fT3/fT4 ratio, SII, SIRI and N/L: low versus high (cutoff median) and SMI (sarcopenic vs. not, cutoff 34 cm^2^/m^2^). With respect to SMI calculation, muscle area at the level of the third lumbar vertebra (L3) was calculated using the 3D Slicer software (version 5.6.1) based on radiological imaging obtained by CT scans.

## 3 Statistical Analysis

Given the exploratory nature of this study, no formal sample size calculation was performed. The sample size was determined pragmatically based on feasibility considerations and aimed to generate preliminary data and hypotheses for future confirmatory studies.

OS and PFS were calculated from the date of initiation of cemiplimab therapy until disease progression or death from any cause.

PFS and OS were the primary outcome measures and estimated using the Kaplan–Meier method and compared with the log‐rank test.

Optimal cutoff of fT3/fT4 ratio for OS and PFS prediction was identified by means of maximally selected rank statistics (MSRS) [21]. PFS and OS time were reported as median survival times with their respective 95% confidence interval (95% CI). ORR was estimated as a percentage. Univariate and multivariate cox‐regression analyses were carried out to assess the independent association between candidate markers and survival.

Associations between inflammatory markers, SMI and fT3/fT4 ratio dichotomised as high versus low were assessed by means of ROC curve analyses and Mann–Whitney tests. The prevalence of categorical variables among patient subgroups was compared using the odds ratio test. Association between SMI and fT3/fT4 was also qualitatively explored by means of scatter plot.

Statistical significance was set at 0.05 probability level for all the tests. All the statistical analyses were performed using the statistical software R version 4.3.0.

## 4 Results

Out of 135 patients included in the previous publication of MITO 44 [6], data on fT3/fT4 ratio were available for 109 patients; eight were excluded for thyroidal comorbidities. Characteristics of the patients were similar to those included in the MITO 44 study (Table 1).

**TABLE 1:** Characteristics of the patients in the MITO 44 and in the present study.

Characteristic	MITO 44 *N* = 128	Present study *N* = 109
Age at the diagnosis		
Median [range]—year	50 [42, 57]	48 [42, 56]
Histologic type		
Squamous cell carcinoma	90 (77.3%)	79 (72.5%)
Adenocarcinoma	24 (18.8%)	19 (17.4%)
Adenosquamous carcinoma	7 (5.5%)	7 (6.4%)
Other	7 (5.5%)	4 (3.7%)
HPV		
Yes	51 (39.8%)	45 (41.3%)
No	19 (14.8%)	16 (14.7%)
Not reported	58 (45.3%)	47 (43.1%)
ECOG performance‐status score		
0	62 (48.4%)	39 (56.9%)
1	42 (32.8%)	12 (11.0%)
2	24 (18.8%)	12 (11.0%)
FIGO stage at diagnosis		
I–II	40 (31.3%)	36 (33.0%)
III–IV	89 (69.5%)	68 (62.4%)
fT3/fT4		
Median [range]	0.25 [0.21, 0.29]	0.27
Haemoglobin value at diagnosis		
Normal range	111 (84.7%)	91 (83.5%)
	14 (10.7%)	13 (12.0%)
PDL1 value		
1%	26 (20.3%)	22 (20.2%)
September	7 (5.5%)	4 (3.7%)
Not reported	95 (74.2%)	74 (67.9%)
Surgery at diagnosis		
Yes	53 (41.4%)	39 (35.8%)
No	75 (58.6%)	63 (57.8%)
Chemotherapy at diagnosis		
Yes	96 (75.0%)	78 (71.5%)
No	32 (25.0%)	26 (23.8%)
Radiotherapy at diagnosis		
Yes	84 (65.6%)	65 (59.6%)
No	44 (34.3%)	38 (34.9%)
Other cases of cervical tumour in the family		
Yes	7 (5.5%)	4 (3.7%)
No	121 (94.5%)	99 (90.8%)
Comorbidity		
Yes	54 (42.2%)	45 (41.3%)
No	74 (57.8%)	59 (54.1%)
Diabetes	10 (7.8%)	7 (6.4%)
Hypertension	30 (23.4%)	27 (24.8%)
Heart disease	8 (6.2%)	6 (5.5%)
Immunologic disease	7 (5.5%)	7 (6.4%)
Others	29 (22.7%)	22 (20.2%)
Prior bevacizumab use		
Yes	75 (56.2%)	60 (55.0%)
No	56 (43.8%)	43 (39.4%)

Abbreviations: FIGO, International Federation of Gynaecology and Obstetrics; HPV, human papilloma virus; PDL1, Programmed Death Ligand 1; yr, year.

Of those, complete blood count (CBC) values were available for 89 patients for calculating SII, SIRI and N/L. Baseline CT scans from 25 patients were available for SMI calculation. At a median follow‐up of 6.9 months, median OS and median PFS of patients included in the present analyses were 15.4 and 4.2 months respectively (Table 2).

**TABLE 2:** Comparison of cemiplimab nominal use in Italy (MITO 44) with the present study.

	MITO 44 (*N* = 128)	Present study (*N* = 109)
mPFS (months)	4.0 (range 3.0–6.0)	4.2 (range 3.2–5.2)
mOS (months)	12.0 (range 12.0, NR)	15.4 (range 9.3–21.5)
CR or NED (*n*, %)	11 (8.6%)	10 (34.0%)
PR (*n*, %)	27 (21.1%)	24 (22.0%)
SD (*n*, %)	19 (14.8%)	15 (13.8%)
PD (*n*, %)	57 (44.5%)	45 (41.3%)
Not evaluable (*n*, %)	14 (10.9%)	10 (34.0%)

Abbreviations: CR, complete response; *N*, number; NED, no evidence of disease; OS, overall survival; PD, progressive disease; PFS, progression‐free survival; PR, partial response; SD, stable disease.

**FIGURE 1:**
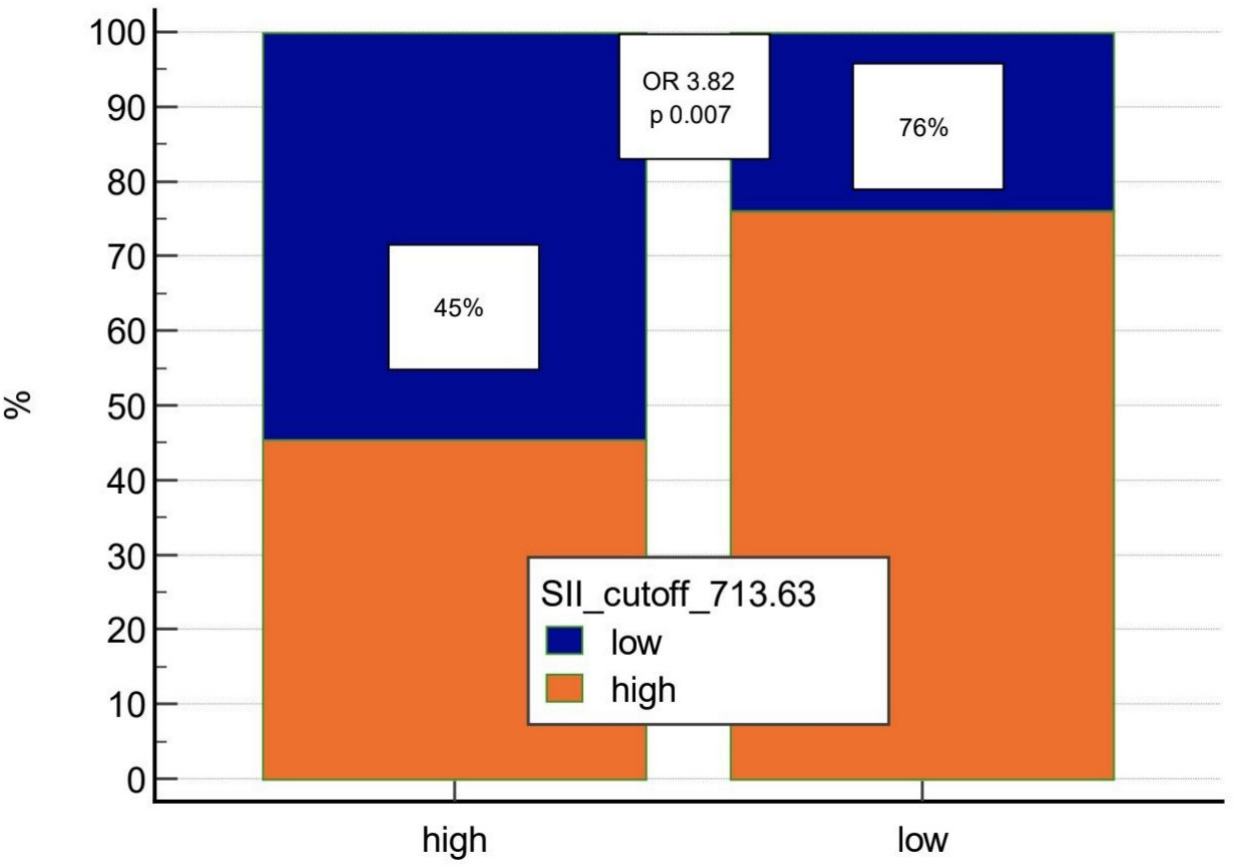
SII value (high vs. low) according to fT3/fT4 ratio high (> 0.29) versus low (≤ 0.29).

Adopting the MSRS approach, the optimal cutoff point for fT3/fT4 values to predict OS was estimated at 0.29; therefore, fT3/fT4 levels below or equal to 0.29 were defined as ‘low’ and levels above 0.29 were defined as ‘high’. Optimal SII cutoff for predicting fT3/fT4 levels was set at 714, adopting a standard ROC curve analysis. Among all possible tested associations, SII showed the strongest correlation with fT3/fT4 levels, OR 3.82 (95% CI 1.39 to 10.50), *p* = 0.0092 (Figure 1).

We performed a mediation analysis to assess whether systemic inflammation (SII) mediates the effect of the fT3/fT4 ratio on OS. The analysis revealed that SII accounted for approximately 8.1% of the effect of fT3/fT4 on OS; however, this indirect effect was not statistically significant (*p* = 0.396). These results support our hypothesis that, although fT3/fT4 and systemic inflammation are correlated, systemic inflammation does not fully explain the impact of fT3/fT4 on OS. This suggests that the prognostic role of fT3/fT4 is, at least in part, independent of the degree of systemic inflammation.

Levels of SII according to fT3/fT4 were also explored by means of Mann–Whitney test for independent samples (Suppl. Figure S1); median SII levels for fT3/FT4 low versus high were 1063 (95%CI 867 to 1387) and 707 (95% CI 521 to 1099) respectively, *p* = 0.066.

An exploratory analysis limited by the small sample size of the subgroup with available SMI data suggested lower SMI values (median = 29.9 cm^2^/m^2^) in a subgroup of very low fT3/fT4 (< 0.20) as compared with higher SMI values (median = 41.4 cm^2^/m^2^) in patients with fT3/fT4 ≥ 0.20 (Mann–Whitney test, *p* = 0.034).

**FIGURE 2:**
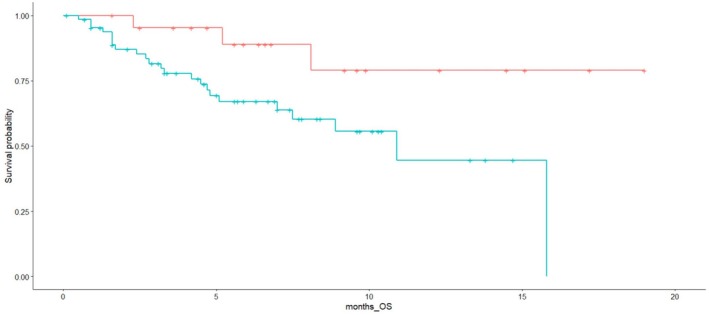
Overall survival curves for patients with low (≤ 0.29, blue line) versus high (> 0.29, red line) fT3/fT4 ratio values.

At univariate analyses, median OS for low versus high SII was not reached (NR) versus 10.9 months (HR 2.15; 95% CI 0.9–4.93, *p* = 0.07), median OS was 10.9 months for patients with low fT3/fT4 while was NR for patients with high fT3/fT4 levels (HR = 2.70; 95%CI 1.17–6.22 *p* = 0.02) (Figure 2).

As expected, patients with ECOG PS 0–1 versus 2 had greater life expectancy 15.8 versus 4.3 months (HR 0.46; *p* < 0.001). At multivariate analysis, both fT3/fT4 ratio and ECOG PS retained their significant impact on OS.

At univariate analyses, median PFS for low versus high SII was 3.7 versus 4.5 months (HR 0.90; 95% CI 0.52–1.57, *p* = 0.72), median PFS was 3.7 months for patients with low fT3/fT4 while was 5.1 months for patients with high fT3/fT4 levels (HR = 1.15; 95%CI 0.65–2.05 *p* = 0.61); patients with ECOG PS 0–1 versus 2 had greater life expectancy 4.4 versus 2.5 months (HR 0.34; *p* = 0.007) (Table 3).

**TABLE 3:** Univariate analysis.

Overall survival				
Pts characteristic		Median (months)	HR (95% CI)	*p*
ECOG PS	0–1	15.8		
	2	4.5		
			0.11 (0.04–0.32)	0.001
SII	Low	NR		
	High	10.9		
			2.15 (0.90–4.93)	0.07
fT3/fT4 ratio	Low	10.9		
	High	NR		
			2.70 (1.17–6.22)	0.02

Progression‐free survival				
Pts characteristic		Median (months)	HR (95% CI)	*p*
ECOG PS	0–1	4.4		
	2	2.5		
			0.34 (0.15–0.75)	0.007
SII	Low	3.7		
	High	4.5		
			0.90 (0.52–1.57)	0.72
fT3/fT4 ratio	Low	3.7		
	High	5.1		
			1.15 (0.65–2.05)	0.61

Abbreviations: CI, confident interval; ECOG, Eastern Cooperative Oncology Group); NR, not reached; SII, systemic inflammation index.

No associations with other variables, such as BMI, nor prognostic effects were found for SIRI, N/L or SMI, the latter limited by low numbers.

## 5 Discussion

The present study demonstrates the prognostic relevance of the fT3/fT4 ratio in patients with aCC treated with cemiplimab. A low fT3/fT4 ratio—indicative of decreased circulating levels of the biologically active hormone T3—has been increasingly recognised across a range of malignancies (including colorectal, renal, urothelial and hepatocellular carcinomas) as a marker of unfavourable prognosis. Our findings suggest that this ratio may reflect early metabolic alterations linked to both tumour aggressiveness and systemic derangements such as pre‐cachexia and sarcopenia, thus offering a potential tool for early identification of patients at higher risk of poor outcomes.

The prognostic value of the fT3/fT4 ratio lies in its ability to signal impaired peripheral conversion of T4 to T3, a process governed by deiodinase enzymes. The incorporation of this biomarker into future multivariate prognostic models could refine patient stratification in clinical trials and optimise therapeutic decision‐making. However, the retrospective design and relatively small sample size limit the generalisability of our results and call for validation in larger, prospective cohorts to fully assess its clinical utility and actionability.

We observed a correlation between the fT3/fT4 ratio and the systemic immune‐inflammation index (SII), a well‐established surrogate of systemic inflammation. This association is biologically plausible, as inflammatory cytokines (such as IL‐1β and IL‐6) have been shown to downregulate the expression and activity of type 1 deiodinase (D1), which is responsible for converting T4 into T3 in the liver and kidneys. Additionally, type 2 deiodinase (D2), primarily active in the brain and brown adipose tissue, is responsible for local tissue‐specific activation of thyroid hormone signalling. Inflammatory mediators and uremic toxins may directly inhibit deiodinase activity, contributing to a vicious cycle of inflammation, metabolic impairment, and reduced thyroid hormone signalling.

Our exploratory analysis also suggested a potential association between low fT3/fT4 ratios and reduced SMI, a recognised indicator of sarcopenia. This finding, although limited by the small number of patients with available imaging data, is consistent with prior observations in non‐oncologic populations. T3 plays a fundamental role in muscle maintenance by regulating gene expression involved in protein synthesis and muscle regeneration. Its deficiency may thus contribute to the development of sarcopenia. However, due to the limited sample size and retrospective nature of the data, no definitive conclusions can be drawn. Similar limitations may have also affected the analysis of correlations with other parameters such as body mass index (BMI).

Moreover, CC may not represent the most appropriate model to study sarcopenia‐related outcomes, given the relatively lower prevalence of clinically significant muscle wasting compared with other tumours. Future studies should explore these associations in settings with a higher burden of sarcopenia, such as gastrointestinal, lung or head and neck cancers.

Despite these limitations, the MITO 44 study provides valuable real‐world data in the context of an emerging therapeutic scenario, with patients treated with cemiplimab—a novel anti‐PD‐1 agent showing efficacy in both clinical trials and routine practice. In this evolving landscape, biomarkers such as the fT3/fT4 ratio, which integrate metabolic and immune dimensions, may offer novel opportunities for risk stratification and personalised intervention strategies.

Emerging evidence suggests that modifiable lifestyle factors, including nutritional status and physical activity, can influence the fT3/fT4 ratio. As such, personalised dietary and exercise interventions might complement immunotherapy by restoring metabolic homeostasis and supporting immune function. This approach could be particularly relevant in the context of ICIs, which have become standard of care across various stages of CC.

Cemiplimab has demonstrated improved overall and PFS in the EMPOWER‐CERVICAL 1 trial, findings that have been mirrored in real‐world data from the MITO 44 study. Other ICIs, such as pembrolizumab and atezolizumab, have shown similar benefits, either as monotherapy or in combination with chemotherapy and radiotherapy, as seen in the KEYNOTE‐826, KEYNOTE‐A18 and BEATcc trials. The earlier integration of immunotherapy appears to yield greater benefit, and ongoing research is exploring its use in increasingly early disease settings.

In this context, the identification and validation of novel prognostic biomarkers—including the fT3/fT4 ratio—are crucial to refine patient selection, optimise treatment strategies and design future clinical trials.

In conclusion, our study supports the prognostic role of the fT3/fT4 ratio in aCC patients treated with cemiplimab and provides preliminary evidence of its association with systemic inflammation and sarcopenia. Further prospective studies are needed to validate these findings and to evaluate whether this biomarker could guide supportive interventions and enhance the effectiveness of immunotherapy.

## 6 Conclusions

The approval of ICIs for both locally advanced and metastatic/recurrent CC, along with ongoing trials exploring their use in earlier stages of disease, is reshaping the therapeutic landscape of CC. As ICIs become increasingly integrated into standard care, there is a growing need to identify and validate additional prognostic markers that can support treatment personalisation.

Our findings suggest that a central biomarker such as the fT3/fT4 ratio may offer valuable prognostic insights. SMI‐related findings from our study should be considered hypothesis‐generating due to their exploratory nature and the limited sample size. These preliminary results highlight the need for validation in larger, prospective cohorts. Moreover, the inclusion of detailed assessments of nutritional status—currently lacking in this dataset—would provide important complementary information and should be prioritised in future studies.

Overall, integrating metabolic, inflammatory and nutritional markers into clinical research may pave the way for more precise and individualised treatment strategies in CC.

## Ethics Statement

This study was approved by Ethical Committee ‘COMITATO ETICO INTERAZIENDALE A.O.U. CITTA’ DELLA SALUTE E DELLA SCIENZA DI TORINO ‐ A.O. ORDINE MAURIZIANO DI TORINO – A.S.L. CITTÀ DI TORINO’, protocol number 0051214 del 21/04/2023. The study was performed in accordance with the Declaration of Helsinki.

## Consent

All the patients signed informed consent.

## Conflicts of Interest

Valentina Tuninetti: honoraria from MSD Oncology, GSK and EISAI; Elisa Virano: none declared; Amedeo Calvo: none declared; Vittoria Carbone: none declared; Carmela Pisano: advisory board: AstraZeneca, MSD Oncology and GSK; Monica Ducceschi: none declared; Giacinto Turitto: none declared; Giuseppa Scandurra: none declared; Maria Cristina Petrella: honoraria from Astrazeneca, MSD and GSK; Valeria Forestieri: none declared; Massimo Petracchini: none declared; Alessandra Bianco: none declared; Raffaella Cioffi: none declared; Mara Mantiero: none declared; Eleonora Paluzzi: none declared; Maria Grazia Distefano: none declared; Olga Martelli: none declared; Sandro Pignata: research funding: AstraZeneca, MSD Oncology, Roche, GSK and Pfizer; honoraria: AstraZeneca, MSD Oncology, Roche, GSK, Novartis, EISAI and PharmaMar; Vittorio Formica: none declared; Fotios Loupakis: none declared; Giorgio Valabrega: consulting fees from GSK; honoraria from AstraZeneca, GSK and MSD; travel support from AstraZeneca and PharmaMar; participation in advisory boards for AstraZeneca, EISAI, GSK and MSD.

## Supporting information


**Figure S1:** SII value distribution among fT3/fT4 ratio, low ratio on the left versus high ratio on the right.

## Data Availability

Etterei no se non obbligatorio.
