# Supplementary material for: Peripheral Thyroid Hormones, Inflammatory and Skeletal Muscle Indexes in Advanced Cervical Cancer Treated With Cemiplimab
Source: J Cachexia Sarcopenia Muscle. 2025 Oct 13;16(5):e70101. doi: 10.1002/jcsm.70101 (PMC12516151; doi:10.1002/jcsm.70101)

**Supplementary Figure 1**. SII values distribution among fT3/fT4 ratio, low ratio on the left vs high ratio on the right.


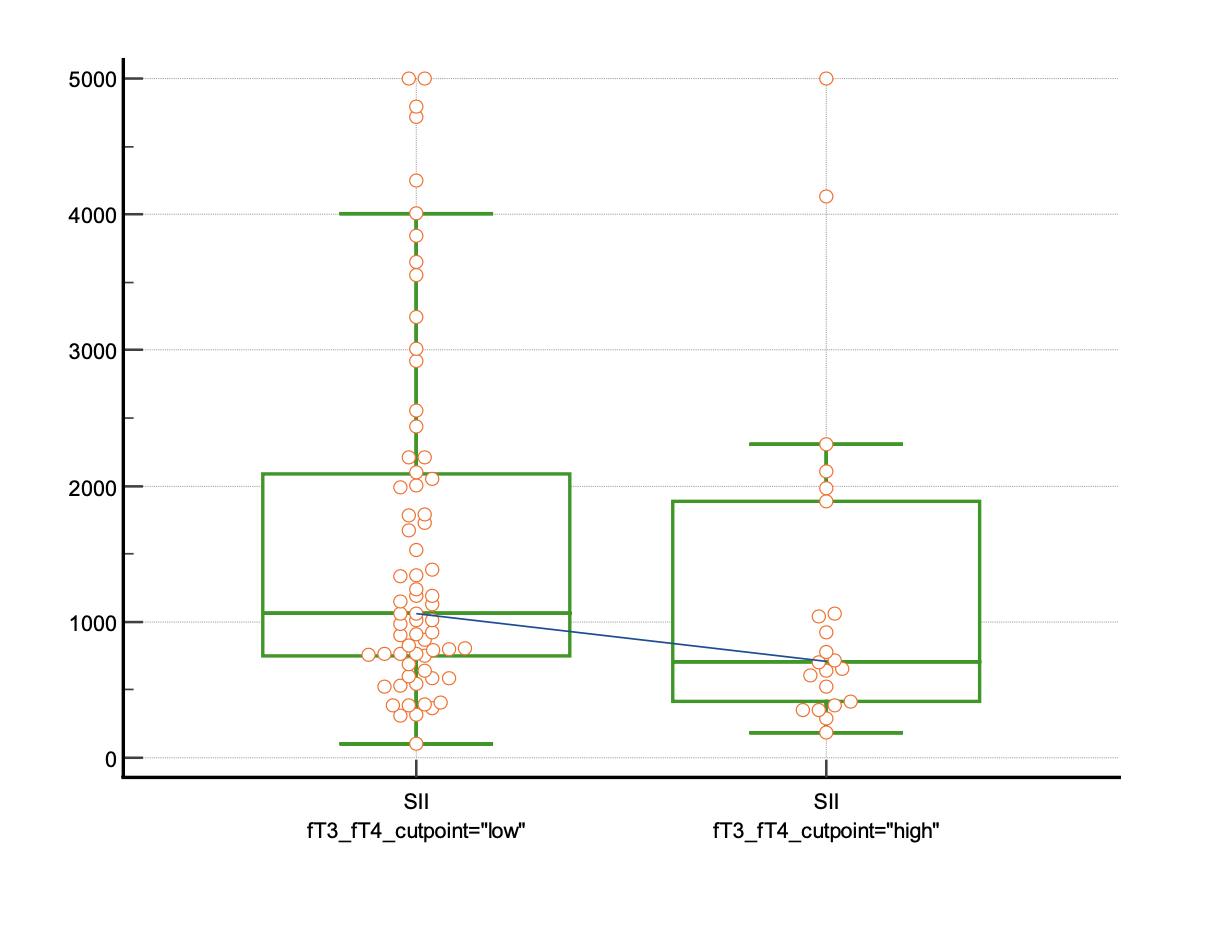

Supplement: Supplementary file 1 — Figure S1: SII value distribution among fT3/fT4 ratio, low ratio on the left versus high ratio on the right. [file JCSM-16-e70101-s001.docx]
